# Advanced Clinical Practitioners' Resilience and Emotional and Spiritual Well-Being During COVID-19

**DOI:** 10.1155/jonm/8892903

**Published:** 2024-12-05

**Authors:** Melanie Rogers, Angela Windle, Lihua Wu, Vanessa Taylor, Chris Bale

**Affiliations:** ^1^Department of Nursing, University of Huddersfield, Huddersfield, UK; ^2^Department of Nursing, Kingston University, London, UK

**Keywords:** advanced clinical practice, COVID-19 pandemic, emotional well-being, resilience, spirituality well-being

## Abstract

**Aim:** This study investigated the impact of the COVID-19 pandemic on the emotional and spiritual well-being and the resilience of advanced clinical practitioners in the United Kingdom.

**Background:** Advanced clinical practitioners are experienced healthcare professionals educated to a master's level who demonstrate expertise, professional judgment, and autonomy across four pillars of advanced practice. Normally, in nursing and the allied health professions, advanced clinical practitioners provide clinical leadership and improve clinical continuity by providing high-quality care to patients through complex decision-making and managing risk. The role contributes to workforce transformation enabling organizations to meet changing population, patient, and service delivery needs. Advanced clinical practitioners' well-being and resilience were particularly at risk during the pandemic due to the increased workload, moral distress, redeployment into other clinical areas, and isolation. Phase 1 of this study identified that advanced clinical practitioners had worryingly low levels of well-being and resilience during the first 6 months of the pandemic. This paper reports Phase 2' findings 1 year into the pandemic.

**Method:** Three hundred and seventy-one respondents completed an online survey comprising three validated scales assessing resilience and emotional and spiritual well-being.

**Results:** One year into the pandemic, advanced clinical practitioners reported a continued decline in their well-being, with average scores on this measure being 12 percent lower compared to prepandemic levels Differences also emerged in the scores of advanced clinical practitioners practicing in primary and secondary care services.

**Conclusion:** Our findings showed the ongoing deleterious impact of the pandemic on the well-being and resilience of advanced clinical practitioners. As the attention of healthcare leaders shifts to the delivery of services post-COVID-19, the longer-term impact of the pandemic on the mental health and well-being of the workforce, alongside the ongoing workforce crisis in the UK and globally, means the well-being and resilience of advanced clinical practitioners need urgent addressing if these role holders are to continue to lead patient care, workforce transformation, and service innovation. Tailored interventions to support advanced clinical practitioners appear necessary to prevent significant workforce impact including absenteeism, long-term stress, sickness absence, and loss to the healthcare workforce.

## 1. Background

The healthcare emergency of the COVID-19 pandemic has challenged global health systems to rapidly adapt to uncertain and dynamic circumstances. Many aspects of the coronavirus were unknown and, as such, posed an additional threat to the health and safety of the healthcare workforce. At the time of writing (13/10/24), there have been over 7,068, 677 deaths globally from the COVID-19 pandemic (World Health Organization [1]). WHO estimated that between 80,000 and 180,000 health and social care workers could have died from COVID-19 in the period between January 2020 and May 2021 [2]. Data from the United Kingdom (UK) Office for National Statistics [3] record 883 deaths involving COVID-19 among health and social care workers (aged 20–64 years) in England and Wales, registered between 9th March and 28th December 2020. These figures have not been updated and are likely to underreport the burden of mortality for this group to date.

The unprecedented physical, moral, and mental health burden experienced by frontline health staff during the first wave of the pandemic has also been reported, including the impact on emotional and spiritual well-being and resilience [4–7]. Studies highlight the impact on well-being when caring for COVID-19 patients on frontline healthcare professionals, including posttraumatic stress, psychological distress, mental exhaustion, anxiety, and depression [8, 9]. A rapid review undertaken in April and May 2020 reported the early impact of COVID-19 on the well-being of healthcare workers [10] and identified the role that occupational and environmental factors in the workplace played as both risk factors and protective factors for mental health outcomes. Review data from Billings et al. [11], focusing predominantly on frontline hospital staff, reported that occupational stress and burnout were closely associated with the fear and the risk of infection, use of personal protective equipment (PPE), social isolation, and feelings of uncertainty that frontline healthcare professionals experienced. Emotional distress and burnout were exacerbated when there was insufficient emotional support, inadequate safety measures, or insufficient institutional support in the working environment. In contrast, training about COVID-19, adequate PPE, and a supportive work environment were protective factors for mental health [11].

These reports reinforce the need to understand the longer-term mental health and well-being impact on health workers throughout, and post, COVID-19 pandemic and to implement and evaluate strategies which aim to mitigate and promote their well-being and strengthen their psychological resilience [10].

The aim of this paper is to report on the quantitative findings from Phase 2 of a longitudinal study investigating the emotional and spiritual well-being and resilience of advanced clinical practitioners (ACPs) in the UK. The study identified that working during COVID-19 continued to impact the emotional and spiritual well-being and resilience of ACPs in the UK.

ACPs have played an integral role in caring for patients with COVID-19 across the globe [12]. ACPs are experienced healthcare professionals educated to master's level and have demonstrated expertise, professional judgment, and their ability to work autonomously across four pillars of advanced practice, clinical, education, research, and leadership (Health Education England [13]). In nursing and the allied health professions, ACPs provide clinical leadership and improve clinical continuity by providing high-quality care to patients through complex decision-making and managing risk as part of a multiprofessional team. Within the UK, the role is contributing to workforce transformation enabling organizations to meet changing population, patients, and service delivery needs [13].

Despite their level of practice and clinical expertise, many ACPs reported that they were not able to “embrace the challenge of COVID-19” and practice to their full scope of practice [14]. More than half the ACPs (52% of *n* = 1000) reported feeling their skills were being underutilized due to factors including a lack of understanding in their organizations about the ACP's role and the potential skills practitioners could provide to patients and the wider organization. In addition, limitations were created by statutory regulation of some professional roles [14]. The underutilization of the ACP role led to frustration and significant concern raised by postholders. Failing to utilize the full potential of the advanced practice role during the pandemic may have contributed to the low levels of well-being and resilience of postholders and to their risk of moral distress as reported by Rogers et al. [5]; Rogers et al. [6] and Wood et al. [7].

Conceptualizations of resilience vary in the literature with early definitions identifying resilience as an individual trait which people either have or do not have. Later theories consider that resilience can be learned and is modifiable. The cyclic resilience development model [15], for example, identified resilience as a resource that individuals can draw on to cope more effectively during stressful situations (positive adaptation) and to use the situation as a learning experience to restore and strengthen their biopsychosocial–spiritual well-being and reduce vulnerability to future stress through greater resilience (cognitive transformation).

Spirituality has been described as having a protective impact on resilience, spirituality embraces issues of meaning, purpose, and hope [16]. Healthcare workers who are aware of their own spirituality and have higher levels of spiritual well-being are likely to be more resilient [17]. More recently, Curtin, Richards, and Fortune [18] presented a multidimensional approach to resilience to connect the internal individual systems and external social and contextual protective and promotive systems to examine health workers' resilience. This approach to resilience explored both the capacity of individuals to navigate their way to the psychological, social, cultural, and physical resources that sustain their well-being and their capacity to negotiate for these resources to be provided and experienced in a culturally meaningful way. By synthesizing qualitative data focused on what promotes resilience in health workers, Curtin, Richards, and Fortune conceptualized that resilience is mainly born out of their professional identity, collegial support, and effective communication from supportive leaders along with flexibility to engage in self-care and experiences of growth [18]. These definitions and conceptualizations, which indicate that a relationship exists between resilience, biopsychosocial–spiritual well-being, and professional role and relationships, clinical context and experiences informed that this study focused on ACPs.

## 2. Methods

In 2020, Phase 1 of this study investigated ACP's emotional and spiritual well-being and resilience during the COVID-19 pandemic. The quantitative findings from Phase 1 are reported in Rogers et al.'s study [6]. Phase 2 was undertaken in 2021 and aimed to explore ACPs' well-being and resilience further on in the COVID-19 pandemic. Quantitative data and demographics are reported. Criteria from STROBE were followed and applied during the study to improve quality and transparency in reporting the findings, and the criteria were applied in all aspects of the study.

### 2.1. Design and Data Collection

As with Phase 1, three validated scales, which are widely used in research, were used to assess the emotional and spiritual well-being and resilience of ACPs. These scales were the Connor–Davidson Resilience 10 Scale (CD-RISC 10), Functional Assessment of Chronic Illness Therapy (FACIT-12)–Spiritual Well-Being 12-Item Scale, and Warwick–Edinburgh Mental Well-Being Scale (WEMWBS) which are used together to provide a holistic view of well-being and resilience.

The WEMWBS was used to assess emotional well-being. Whilst the scale was originally designed to measure emotional well-being in the general population [19], it has been used extensively in empirical studies. Fourteen Likert-scale questions specifically focus on the thoughts and feelings of participants related to their well-being. Scoring is standardized. The higher the score, the greater the emotional well-being participants report. Lower scores, specifically under 42 relate to a reduced level of emotional well-being. The scale has been extensively used and validated across a range of populations [20], and demonstrates reliability, with a high level of internal consistency in our sample (Cronbach's *α* = 0.91).

To measure spiritual well-being, the FACIT-12 (Version 4)–Spiritual Well-Being Scale was used. There are several functional assessments of chronic illness scales which are validated [21]. These scales include measures on a number of aspects of well-being including social, spiritual, and physical well-being that can be utilized in participants over the age of 18. Version 4 of this scale is validated and has been adapted for use with healthcare workers. Likert scoring is incorporated in the eight scale questions which relate participants' responses to feeling a sense of peace or meaning in their lives. Four further questions focus on faith. A manual scoring guide has been developed for FACIT-12 which was utilized during data analysis, and some items are reverse scored. Those with light spiritual well-being have a higher FACIT-12 score. Of interest, respondents who do not identify with having a “faith” but do identify as “spiritual” are able to report aspects of the scale, for example, meaning, peace, and faith. Internal reliability is demonstrated (Cronbach's *α* = 0.69).

The CD-RISC measures resilience levels and was initially designed for use with participants who had a diagnosis of posttraumatic stress disorder utilizing a scale with 25 questions [22]. Connor and Davidson [22] added a shorter 10-question scale after factor analysis. It has been utilized and validated in numerous settings by many health professionals [22]. Likert scales with 10 questions focus on related psychological resilience and trait resilience. Higher resilience is identified with higher scores. Internal reliability was demonstrated in this sample (Cronbach's *α* = 0.89).

Permission was gained by the authors to utilize all three scales prior to data collection.

A Qualtrics survey was developed which incorporated all three scales. The survey included demographic questions which included age, gender, years of ACP experience, and level of education. We also sought data related to where participants worked, what hours they worked, and what professional title they used in practice. Further data were collected via open responses which were analyzed qualitatively and reported elsewhere. The survey was open from July to October 2021.

Invitation to participate in the study was through personal and professional advanced practice networks of the research team throughout the UK. As snowball sampling was employed by asking recipients to share with colleagues and networks, it is impossible to estimate how many ACPs received the survey. Additional invitations were sent to participants from Phase 1 of the study if they had shared their email addresses. All responses were anonymized. Inclusion criteria ensured participants were

• Working in the UK as an ACP according to the definition of Health Education England [13]: OR
• An ACP who had attained a credential from the national government or by a royal college, for example, the Royal College of Nursing: OR
• Undertaking their MSc Advanced Clinical Practice education.

The University of Huddersfield (SREIC 2021/043) granted ethical approval.

### 2.2. Data Analysis

Three hundred and seventy-one surveys were completed. In total, 85 participants (22.9%) had one or more items of missing data. However, the only variables where more than five percent of participants had missing data were those asking about experiences with COVID-19 and the number of hours participants worked per week. Missing values were not imputed, and participants with any missing data for a given variable were excluded from any analyses involving that variable.

The selection of variables was based on their relevance to the study aim and Phase 1 of the study [6]. All data analysis was conducted using IBM SPSS Version 29 statistical software. Descriptive statistics were calculated for all study variables and are presented in Tables 1 and 2. To assess the overall level of emotional well-being, that is, the total WEMWBS scores in the current sample, a one-sample *t*-test comparing this with a previous general population sample taken from the Health Survey for England [23] was conducted, and the size of this difference was assessed using Hedges' g [24]. This procedure was also used to compare emotional well-being and resilience (CD-RISC-10) in our sample to a similar sample of ACPs we surveyed earlier in the pandemic from July to August 2020 [6].

A number of one-way ANOVAs were conducted in order to examine whether levels of emotional well-being, resilience, overall spirituality (FACIT-SP-12), faith, and meaning differed between participants working in different clinical roles and settings and with different levels of highest qualification. Independent samples *t*-tests were conducted to examine whether there were differences in well-being, resilience, or spirituality between genders, or participants who had directly cared for those with COVID-19, had experience of caring for patients in previous pandemics, had been redeployed during the pandemic, and had been diagnosed with COVID-19 themselves or had family members or colleagues who had, in comparison to those who had not.

A stepwise linear multiple regression analysis was conducted to examine potential predictors of emotional well-being in the current sample. In particular, whether resilience and spirituality predicted emotional well-being after controlling for other relevant variables. In the first step, the continuous variables age, the number of hours per week participants reported currently working, and the number of years they had worked were entered together with several categorical variables. The categorical variables used were as follows: (dummy-coded) sex, whether participants were providing direct care or testing for COVID-19, whether they had previous experience providing care in epidemics, whether they had been redeployed during the pandemic, and whether they, or any of their family or coworkers, had been diagnosed with COVID-19. In the second step, resilience, faith and meaning were entered as additional predictors. Since these were the predictors of most interest in the current study, these were entered in this separate second step in order to assess whether they explained any additional variance in emotional well-being over and above the variables which were controlled for in the first step. A similar analysis was conducted to examine potential predictors of resilience, following the same procedure, with the exception that in the second step, faith and meaning were entered as predictors. In order to examine potential predictors of overall spirituality, faith, and meaning, the predictor variables described above for the first step were simultaneously entered into a single multiple regression model for each of these outcomes.

The alpha level for all analyses was set at 0.05, and any results with *p* values lower than this were considered to be statistically significant.

## 3. Results

Table 1 shows the number of participants in different demographic and occupational groups in our sample. Our participants were aged between 25 and 67 (*M* = 44.8, SD = 9.2) and had worked as ACPs/advanced practice nurses (APNs) for up to 23 years (*M* = 5.0, SD = 4.7) and worked for up to 55 h per week (*M* = 34.7, SD = 6.3). They were predominantly (83%) women, and most (55%) were employed in primary care, with the remainder working in secondary care (22%), intensive care, emergency departments and COVID-19 wards (13%), or other settings (10%). The majority (57%) were ACPs, though some were MSc students studying for this role (32%) or identified themselves as nurse practitioners (9%), with relatively fewer clinical nurse specialists (2%). Most (63%) were educated to master's level, with the remainder holding bachelor's degrees (25%), diplomas (6%), doctoral degrees (1%), or other qualifications (5%).

Table 2 shows participants' responses to our questions about their experiences during the pandemic. The majority (65%) were providing direct care or testing for patients with COVID-19, although most (63%) had no experience of providing care in previous pandemics, and a minority (17%) had been redeployed during the pandemic. Twenty-nine percent of participants had been diagnosed with COVID-19, and most (86%) had family members or coworkers who had been diagnosed with COVID-19.

WEMWBS scores for our total sample were significantly lower than population norms taken from the Health Survey for England [23] (*M* = 45.4 vs 51.6, *t* [366] = −14.53, *p* < 0.001). This was a medium to large effect (Hedges' g = 0.72) which suggests that levels of emotional well-being were significantly lower in this ACP sample than in the (pre-COVID-19) general population. However, there were no significant differences in either well-being (*M* = 45.4 vs 45.1, *t* [366] = 0.33, *p*=0.43) or resilience (*M* = 27.7 vs 27.6, *t* [347] = 0.35, *p*=0.73) between our current sample and a similar sample of ACPs we surveyed between July and August 2020 [6]. These findings indicate that the decreased levels of well-being in ACPs we observed during the early months of the pandemic have persisted, though there is no evidence of either an increase or decrease in resilience over the course of the pandemic in this population.

ANOVAs indicated that there was a significant difference in overall levels of mental well-being between ACPs working in different clinical settings (*F* [2, 260] = 3.30, *p* < 0.05), and post hoc tests indicated that those working in intensive care, emergency departments, or COVID-19 wards had significantly lower well-being than those working in primary care (*M* = 42.8 vs 46.1, *p* < 0.05). However, there were no significant differences in resilience or spirituality between different demographic groups (Table 1), demonstrating similar levels across participants in different clinical roles and with different qualifications.

Independent samples *t*-tests indicated that participants who had directly cared or tested for patients with COVID-19 had significantly lower levels of well-being (*M* = 44.4 vs 46.7, *t* [281] = 2.21, *p* < 0.05), overall spirituality (*M* = 25.7 vs 28.5, *t* [273] = 2.46, *p* < 0.05), and meaning (*M* = 19.7 vs 21.6, *t* [274] = 2.42, *p* < 0.05) than those who had not. However, there were no other differences in well-being, resilience or spirituality between participants who had different experiences during the pandemic, and no gender differences on these variables in our sample.

Multiple regression analyses indicated that participant's age, sex, years of experience, hours worked per week, and experiences during the pandemic did not significantly predict their levels of well-being. However, adding spirituality and resilience produced a model which significantly predicted well-being (*F* [12, 218] = 38.97, *p* < 0.001) and accounted for 67% of the variance in this. This indicates that, after controlling for demographic and occupational differences, and participants' experiences during the pandemic, spirituality and resilience strongly predicted emotional well-being (*F* [3,218] = 145.19, *p* < 0.001, *r*-squared change = 0.64). However, whilst the meaning component of spirituality significantly predicted well-being in this final model, the faith subscale did not.

Participants who reported higher levels of resilience also reported greater emotional well-being (*β* = 0.22, *B* = 0.28, 95% CI: [0.16, 0.40], *p* < 0.001). With respect to spirituality, although faith did not predict emotional well-being in our sample, meaning was the strongest predictor of well-being, with participants who reported higher levels of meaning also reporting significantly greater emotional well-being (*β* = 0.66, *B* = 0.88, 95% CI: [0.74, 1.02], *p* < 0.001).

The only significant predictor of resilience was spirituality (*F* [11,222] = 14.14, *p* < 0.001) which accounted for 38% of the variance in resilience (adjusted r-squared = 0.38). Participants with higher levels of meaning reported significantly greater levels of resilience (*β* = 0.65, *B* = 0.70, 95% CI: [0.57, 0.82], *p* < 0.001), though faith did not significantly predict resilience.

The final multiple regression analyses indicated that there were no significant predictors of overall spirituality, faith, or meaning in our sample.

## 4. Discussion

In this paper, we report the quantitative findings of Phase 2 of a study investigating the emotional and spiritual well-being and resilience of ACPs. There are two main significant findings from our Phase 2 study: (1) the continued low well-being scores of ACPs 1 year on from the Phase 1 survey and (2) the impact of clinical area of practice on the ACPs' well-being scores.

During the pandemic, ACPs were called upon to meet the increased demand for healthcare services and were involved in face-to-face and telehealth patient care provision, including triage of patients, remote monitoring and consultations, as well as the direct clinical care of people suffering from COVID-19. ACPs faced challenges in adapting to new technologies and protocols and dealing with the emotional and psychological impact of the pandemic on their patients [6]. Some ACPs also faced workplace challenges, with more than half reporting frustration that the range of capabilities practitioners could provide to patients and the wider organization were not fully understood or utilized by their employers [14].

The ACPs surveyed in the second phase of this research have continued to record low well-being scores (WEMWBS) (*p* < 0.05) and these scores have not improved since Phase 1 of the study [5] at the outset of the pandemic. A resilient practitioner is described as a practitioner with the capacity to navigate their way to the psychological, social, cultural, and physical resources that sustain their well-being and has the capacity to negotiate for these resources to be provided and experienced in a culturally meaningful way [18]. However, the ACPs in our survey do not appear to have been able to return to their prepandemic levels of well-being and resilience when measured against the general UK population and have not been able to bounce back despite any connection with faith, spirituality, or meaning. Brown [25] suggests that spirituality and resilience are deeply connected. As spirituality relates to what gives us hope, meaning, and purpose, it can help to maintain our resilience [6], however, the results show that this had not been the case 1 year into the pandemic.

The ACPs surveyed continued to have lower well-being scores compared to the general population (*p* < 0.001) and similar levels of low emotional well-being 1 year later from the initial Phase 1 responses. A number of factors may have contributed to the continuing impact on practitioners' well-being and resilience scores. As identified by Curtin, Richards, and Fortune [18], resilience may be impacted by the challenges to their professional identity, the availability of collegial support, effective communication from supportive leaders, and the flexibility to engage in self-care and experiences of growth. The extent to which these ACPs had access to supportive interventions and resources for their health and well-being or were able to ask for this support is explored in the qualitative feedback and reported separately.

What does appear to have been a significant factor in the ACPs' well-being scores is the area of clinical practice during COVID-19 (*p* < 0.05). The closer the practitioners were at the forefront of the direct care of COVID-19 patients does appear to have been the second significant finding. Notably, the scores for practitioners from a secondary setting, such as the emergency department, critical care, or general COVID-19 wards, were much lower (*p* < 0.5) than their counterparts in a primary care environment. These findings correlate with Wood et al. [7], who surveyed 97 APNs using the short version of the WEMWBS questionnaire [20] to explore their experience of moral distress during the COVID-19 pandemic. Moral distress is described as arising from a situation where a person is unable to undertake the right course of action because of institutional constraints [26]. Wood et al. [7] identified that, whilst APNs in all settings were mentally and physically exhausted, levels of moral distress were significantly higher among APNs working in secondary care compared to those working in primary care. The type of distress and its causes also varied with APNs in secondary care tending to experience trauma and APNs working in primary care tending to report isolation.

A number of authors (for example, [7, 11, 18]) recommend that a flexible system of support involving peer, organization, and professional support is now required to foster resilience at the individual, organizational, and community/contextual levels and involving greater collaboration, consultation, and coproduction of support services with healthcare staff to identify the targeted support they require. The importance of robust evaluation of these strategies is also recommended, including factors that may act as barriers and facilitators to the implementation of interventions within local settings [11, 18, 27].

One approach to support currently being piloted by the research team for ACPs is resilience-based clinical supervision (RBCS), a type of clinical supervision, which has been found to develop an increased awareness of the importance of self-care and question organizational practices which impact negatively on staff (including their own) and patient well-being [28]. RBCS focuses on the “emotional systems motivating the response to a situation” and includes elements of mindfulness-based exercises with a view to “enhancing well-being, resilience, and improving patient care.” In the context of continuing workforce challenges and poor staff surveys, Bailey and West [29] and Docherty [30] assert that in the postpandemic period, there is an opportunity for learning from staff experiences of COVID-19 and to make substantial changes in culture and infrastructure to meet the needs of a workforce changed by what it has been through, including the already depleted workforce reserves which existed prepandemic. Following evaluation, access to RBCS may offer an opportunity to support the emotional and spiritual well-being and resilience of ACPs enabling the strengthening of psychological resilience in a personalized approach, potentially protecting these ACPs from adverse mental health outcomes, alongside structural changes to create a healthy, safe, and supportive work environment [10].

## 5. Conclusion

ACPs working throughout the COVID-19 pandemic continued to have low well-being scores and their resilience was impacted in response to continued stressors, especially those ACPs working at the forefront of direct patient care in hospital settings. To prevent significant workforce impact, including absenteeism, long-term stress, sickness absence, and loss to the healthcare workforce, the need for more nuanced and targeted health and well-being support strategies for those practicing in different services appears warranted to mitigate and promote their well-being and strengthen their psychological resilience, alongside structural and organization changes.

### 5.1. Limitations

The study has several limitations. First, the study design makes it difficult to ascertain any causal relationships from the cross-sectional analysis and is limited by the responses provided. Second, the response rate may have been impacted by the challenges faced in the pandemic. Finally, the findings may not be generalizable due to the lack of standardization of the ACP title and role which could have led to some potential participants feeling ineligible to participate in the study. However, the study has external validity with the different ACP specialties represented and participants from across the United Kingdom.

### 5.2. Implications for Nursing Management

Whilst efforts made to prioritize and provide enhanced health and well-being services were made for the workforce during the COVID-19 pandemic, the study findings emphasize the urgent need to understand the flexible system of support and the targeted interventions required from healthcare systems, peer, organizational, and professional, to better support the well-being and resilience of ACP in different settings. Through collaboration and coproduction, there is also an opportunity to examine the structural, systemic, and individual barriers to accessing psychological and well-being support and ensure that evaluation is undertaken.

## Figures and Tables

**Table 1:** Descriptive statistics for all study measures by demographic group.

Variable	*N* (%)	WEMWBS		CD-RISC		FACIT-SP-12 total		FACIT-SP-12 faith		FACIT-SP-12 meaning	
		M	SD	M	SD	M	SD	M	SD	M	SD
Sex											
Male	62 (17)	44.5	8.7	27.1	6.8	25.6	9.0	5.6	4.3	20.0	6.1
Female	308 (83)	45.7	8.2	27.8	6.3	26.9	8.9	6.5	4.0	20.4	6.1
Title											
Advanced clinical practitioner	210 (57)	46.2	8.6	28.1	6.3	26.8	9.1	6.1	4.1	20.7	6.2
Nurse practitioner	31 (8)	45.2	7.3	26.0	6.4	27.3	10.3	7.3	4.7	20.0	6.9
Clinical nurse specialist	6 (2)	44.5	5.7	26.0	9.9	22.5	7.4	4.0	2.0	18.5	6.0
MSc ACP student	119 (32)	44.4	7.9	27.4	6.6	26.4	8.4	6.5	3.9	20.0	5.7
Other	5 (1)	43.3	7.7	25.3	4.1	25.5	7.7	6.3	2.6	19.3	6.4
Education											
Diploma	22 (6)	45.8	8.4	26.8	8.0	24.6	9.6	5.1	3.8	19.6	6.8
Bachelor's degree	94 (25)	45.1	7.9	26.4	6.3	26.4	8.3	6.7	4.0	19.7	5.5
Master's degree	235 (63)	45.5	8.5	28.1	6.2	26.8	8.9	6.2	4.0	20.6	6.2
Doctoral degree	3 (1)	40.7	3.8	30.3	2.9	27.3	15.8	7.3	8.1	20.0	8.7
Other	17 (5)	47.4	7.3	28.5	8.1	29.4	10.1	7.1	4.5	22.3	6.6
Clinical setting											
Primary care	204 (55)	46.1	8.3	27.9	6.4	27.0	8.9	6.5	4.0	20.5	6.3
Secondary care	81 (22)	45.1	9.2	27.1	6.7	26.6	9.5	6.3	4.3	20.4	6.3
Intensive care/emergency department/COVID-19 ward	49 (13)	42.7	6.5	27.1	4.8	24.7	7.6	5.5	3.8	19.2	4.8
Other	37 (10)	46.2	7.5	28.4	7.3	27.8	9.3	6.7	4.3	21.1	6.0
Total	371	45.4	8.1	27.7	6.4	26.5	9.0	6.3	4.1	20.3	6.2

Abbreviations: CD-RISC, Connor–Davidson Resilience Scale; FACIT-SP-12, Functional Assessment of Chronic Illness Therapy–Spiritual Well-Being 12-Item Scale; WEMWBS, Warwick–Edinburgh Mental Well-Being Scale.

**Table 2:** Experiences with COVID-19, mental well-being, resilience, and spirituality.

Question	*N* (%)	WEMWBS		CD-RISC		FACIT-SP-12 total		FACIT-SP-12 faith		FACIT-SP-12 meaning	
		M	SD	M	SD	M	SD	M	SD	M	SD
Are you providing direct care or testing for patients with COVID-19?											
No	102 (35.5)	46.9	8.9	28.1	6.8	28.4	9.1	6.8	4.2	21.6	6.3
Yes	185 (64.5)	44.4	8.0	27.0	6.4	25.7	8.7	6.0	3.9	19.7	6.1
Have you had experience providing care for patients during previous epidemics (for example, Ebola, SARS, and MERS)?											
No	182 (63)	45.4	7.8	27.3	6.5	26.7	9.0	6.2	4.1	20.5	6.1
Yes	105 (37)	44.9	9.2	27.5	6.6	26.6	8.9	6.4	3.9	20.1	6.4
Were you redeployed during the COVID-19 pandemic?											
No	236 (83)	45.5	8.2	27.4	6.6	26.8	8.7	6.3	3.9	20.5	6.1
Yes	50 (17)	44.2	9.2	27.1	6.4	26.1	10.0	6.5	4.3	19.7	6.6
Have you been diagnosed with COVID-19?											
Yes	203 (71)	45.6	8.4	27.4	6.3	27.1	9.0	6.5	4.1	20.6	6.2
No	83 (29)	44.5	8.2	27.3	7.0	25.5	8.7	5.8	3.7	19.7	6.2
Have any of your family members or your coworkers been diagnosed with COVID-19?											
No	39 (14)	44.7	9.1	28.1	6.7	26.1	9.4	5.9	4.3	20.2	6.6
Yes	248 (86)	45.3	8.2	27.2	6.5	26.7	8.9	6.4	4.0	20.4	6.2

Abbreviations: CD-RISC, Connor–Davidson Resilience Scale; FACIT-SP-12, Functional Assessment of Chronic Illness Therapy–Spiritual Well-Being 12-Item Scale; WEMWBS, Warwick–Edinburgh Mental Well-Being Scale.

## Data Availability

DOI: 10.17632/wpz7tr5fvt.1 Mendelay Data.
